# Sliding-strip microfluidic device enables ELISA on paper

**DOI:** 10.1016/j.bios.2017.07.034

**Published:** 2018-01-15

**Authors:** Mohit S. Verma, Maria-Nefeli Tsaloglou, Tyler Sisley, Dionysios Christodouleas, Austin Chen, Jonathan Milette, George M. Whitesides

**Affiliations:** a Department of Chemistry and Chemical Biology, Harvard University, 12 Oxford Street, Cambridge, MA 02138, USA; b Wyss Institute for Biologically Inspired Engineering, Harvard University, 60 Oxford Street, Cambridge, MA 02138, USA; c Kavli Institute for Bionano Science and Technology, Harvard University, 29 Oxford Street, Cambridge, MA 02138, USA; d Diagnostics for All, 4 Technology Way, Salem, MA 02138, USA

**Keywords:** Resource-limited, C-reactive protein, Blood, Sepsis, Inflammatory bowel diseases, Receiver operator characteristics

## Abstract

This article describes a 3D microfluidic paper-based analytical device that can be used to conduct an enzyme-linked immunosorbent assay (ELISA). The device comprises two parts: a sliding strip (which contains the active sensing area) and a structure surrounding the sliding strip (which holds stored reagents—buffers, antibodies, and enzymatic substrate—and distributes fluid). Running an ELISA involves adding sample (e.g. blood) and water, moving the sliding strip at scheduled times, and analyzing the resulting color in the sensing area visually or using a flatbed scanner. We demonstrate that this device can be used to detect C-reactive protein (CRP)—a biomarker for neonatal sepsis, pelvic inflammatory disease, and inflammatory bowel diseases—at a concentration range of 1–100 ng/mL in 1000-fold diluted blood (1–100 µg/mL in undiluted blood). The accuracy of the device (as characterized by the area under the receiver operator characteristics curve) is 89% and 83% for cut-offs of 10 ng/mL (for neonatal sepsis and pelvic inflammatory disease) and 30 ng/mL (for inflammatory bowel diseases) CRP in 1000-fold diluted blood respectively. In resource-limited settings, the device can be used as a part of a kit (containing the device, a fixed-volume capillary, a pre-filled tube, a syringe, and a dropper); this kit would cost ~ $0.50 when produced in large scale (>100,000 devices/week). This kit has the technical characteristics to be employed as a pre-screening tool, when combined with other data such as patient history and clinical signs.

## Highlights

• 3D microfluidic paper-based analytical device performs ELISA with colorimetric results.
• Two components enable separation of reagents in the device: a sliding-strip and a functional dock.
• All required reagents (antibodies, enzyme, substrate, buffers) are stored in the device.
• User only needs to add sample and water using the provided kit.
• Device can detect C-reactive protein for possible pre-screening of neonatal sepsis, pelvic inflammatory disease, or inflammatory bowel diseases.

## 1 Introduction

Microfluidic paper-based analytical devices (µPADs) (Li et al., 2008, Martinez et al., 2007, Martinez et al., 2008b) are valuable tools for satisfying the World Health Organization's ASSURED (affordable, sensitive, specific, user-friendly, rapid and robust, equipment-free, and deliverable to end-users) (Kettler et al., 2004) criteria for diagnostic devices in developing countries (Cate et al., 2015, Mao and Huang, 2012, Yetisen et al., 2013). They are, however, typically limited to single-step biochemical assays (i.e. a single step of mixing samples and reagents), and thus are unable to perform complex assays—such as enzyme-linked immunosorbent assays (ELISAs)—that require mixing of multiple reagents and removal of excess reagents in an ordered sequence. Here, we circumvent this limitation of µPADs by incorporating a sliding strip (Connelly et al., 2015, Liu et al., 2011) into the device that allows switching among fluidic paths, and makes it possible to contact the sample and the reagents required for a bioassay—capture antibodies, enzyme-linked detection antibodies, and substrate for enzyme—in a timed sequence, and only in the correct order. This project is intended primarily as a demonstration of principle of a sliding-strip device for immunoassays and its accuracy is still short of that required for clinical analysis. It is, however, sufficient for a screening or pre-screening assay.

ELISA is one of the most common methods for detecting and quantifying biomarkers (both proteins and small molecules) (Engvall et al., 1971, Engvall and Perlmann, 1971, Lequin, 2005). As used in a conventional 96-well format, ELISA is not suitable for resource-limited environments because it requires: i) trained personnel, ii) expensive analytical instruments (e.g. a microplate reader), and iii) multiple steps of mixing reagents and washing (these steps are difficult to adapt to a simple and portable device to be used by healthcare workers with limited experience). Multi-well plate formats also assume the need for multiple assays, and are designed for high-volume laboratories; they are thus often inappropriate for single-patient assays at the point of care, or in low-volume clinics. µPADs have the potential to overcome many of these drawbacks because they are: i) lightweight (a few grams), ii) easily fabricated from paper, double-sided adhesive tape, and hydrophobic film, and iii) easily disposed of by incineration (Cate et al., 2015, Mao and Huang, 2012, Martinez et al., 2010b, Yetisen et al., 2013). They operate without the need for external power or equipment (e.g., pumps). In particular, µPADs with complex, 3D microfluidic channels (3D µPADs (Martinez et al., 2010a; Martinez et al., 2008b)) are capable of distributing small quantities (~ 20–100 µL in current designs) of samples from a single inlet into a large array of test zones (in principle, up to thousands (Martinez et al., 2008b)), and permit the multiplexing of array-based assays. They are also suitable for use with single patients at the point of care. We (Cheng et al., 2010, Connelly et al., 2015, Martinez et al., 2007, Martinez et al., 2010a, Martinez et al., 2008b, Pollock et al., 2012, Vella et al., 2012) and others (Abe et al., 2010, Ali et al., 2009, Dungchai et al., 2009, Fu et al., 2010, Khan et al., 2010, Li et al., 2008, Li et al., 2010b, Liu et al., 2013, Noh and Phillips, 2010, Struss et al., 2010, Toley et al., 2013, Yu et al., 2011) have developed µPADs for diagnostic applications, but these devices were generally not equipped to perform ELISA—except for some demonstrations where reagents were simply added to paper instead of to a 96-well plate (Cheng et al., 2010, Hsu et al., 2014). Even in these examples of µPADs for ELISA, the complexity of the assay is maintained because the user is required to dispense multiple reagents (antibodies and substrate for the enzyme).

Here, we describe a portable device for ELISA that contains stored reagents—capture antibody, detection antibody, substrate, and buffers—in isolated zones and requires only the addition of sample and water to complete an assay. The reagents are brought into contact with each other by moving the sliding strip manually to different zones. Ismagilov *et al.* have developed a conceptually similar method for manipulating fluids in glass/plastic microfluidic devices (called “SlipChip” ([Bibr bib3][Bibr bib4]; Du et al., 2009; Li et al., 2010a; Liu et al., 2010; Ma et al., 2014; Shen et al., 2010a [Bibr bib53][Bibr bib54])), where two plates with patterned micro-wells and channels slide relative to one another to form different fluidic pathways and bring reagents in and out of contact (Begolo et al., 2013, Begolo et al., 2014, Du et al., 2009, Li et al., 2010a, Liu et al., 2010, Ma et al., 2014, Shen et al., 2010a, Shen et al., 2010b, Shen et al., 2011). We have also developed a similar method for manipulating fluids using a “paper machine” for molecular diagnostics (loop-mediated isothermal amplification reaction for *Escherichia coli malB* gene), where a magnetic strip moves between layers of magnets, and paper is used as the active reaction matrix (Connelly et al., 2015). We compare our “sliding strip” methodology to Ismagilov's SlipChip and Connelly's paper machine in the Results and discussion section under the title “comparison to similar devices.”

## 2 Materials and methods

### 2.1 Materials and equipment

Please see Supplementary information for details on suppliers for materials and equipment.

### 2.2 Fabrication

**Fig. 1:**
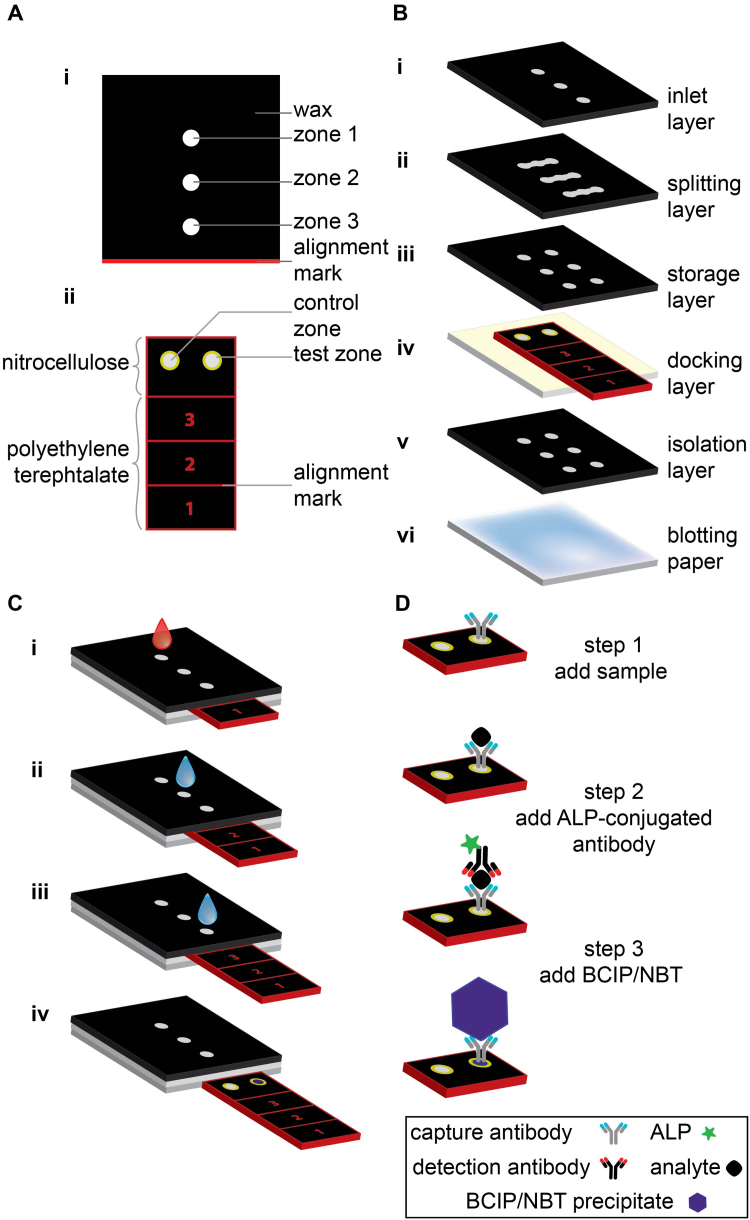
Schematic illustration of the sliding-strip 3D µPAD. A) Top view of the (i) functional dock and (ii) sliding strip showing various parts of each component. B) Components of the sliding-strip 3D µPAD, each layer is glued using double-sided tape with holes that connect the fluidic channels. C) Operation of the sliding-strip 3D µPAD: i) while the sliding strip is in position 1, sample is added to the inlet and washed with water, ii) the sliding strip is moved to position 2 and water is added to the inlet to dissolve the stored detection antibodies and buffer, and to wash off excess detection antibodies, iii) the sliding strip is moved to position 3 and water is added to the inlet to dissolve stored substrate and buffer, and to wash off excess substrate, iv) the sliding strip is removed from the device to analyze the results visually or using a desktop scanner. D) Mechanism of operation of the sliding-strip device, the steps are analogous to those in part C. (For interpretation of the references to color in this figure, the reader is referred to the web version of this article.)

Please see Supplementary information for details on the fabrication of the device (Fig. 1).

### 2.3 Detection

The sample was prepared by mixing 1 µL sheep blood, 1 µL of solution of C-reactive protein (CRP) (at concentrations of 1, 20, 40, 60, 80, and 100 µg/mL), and 998 µL 1% w/v bovine serum albumin (BSA) in phosphate buffered saline (PBS, pH 7.4) to simulate a 1000-fold diluted blood sample. The 998 µL of 1% w/v BSA was measured using a micropipette and pre-aliquoted in tubes, while the 1 µL blood and CRP were measured using fixed-volume capillaries. The capillaries were placed in the tube containing BSA and the tubes were shaken to mix the liquids.

While running the assay, 100 µL of sample (as prepared above) was added to the first hole using a micropipette and allowed to wick into the device and incubated for 30 min at room temperature. Water (100 µL) was added to the first hole to wash off excess sample. The sliding strip was pulled to the second position and 150 µL of water was added to the second hole to elute the detection antibody and to wash off excess antibodies. The sliding strip was pulled to the third position and 150 µL of water was added to the third hole to dissolve the stored 5-bromo-4-chloro-3-indolyl phosphate (BCIP)/nitro blue tetrazolium (NBT) substrate and to wash off excess substrate. The sliding strip was pulled out of the dock and allowed to dry for 30 min under ambient conditions before imaging using a desktop scanner (Epson J251A) to obtain 48-bit Red, Green, and Blue (RGB) images (48-bit images provide a larger dynamic range for a concentration-dependent response than 24-bit images).

When emulating resource-limited settings, the sample (100 µL) was added using a disposable syringe (instead of a micropipette) and water (two drops) was added using a plastic dropper (instead of a micropipette).

### 2.4 Analysis

**Fig. 2:**
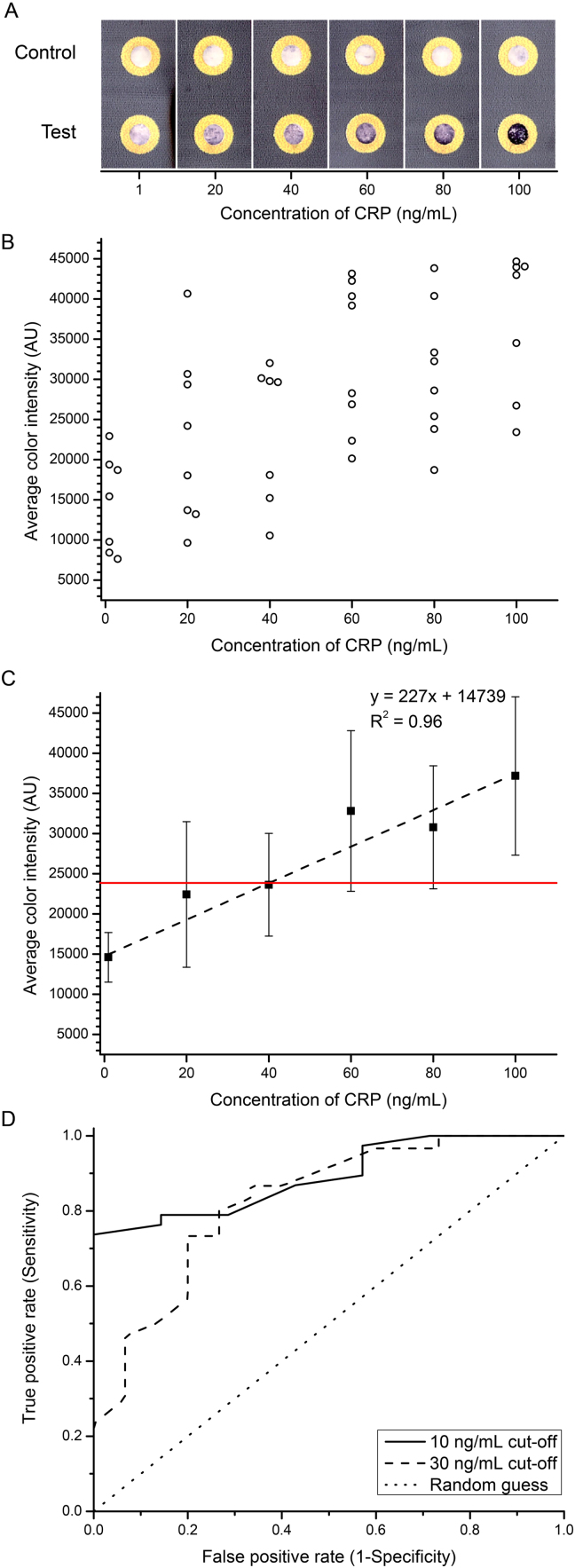
Detection of human C-reactive protein (CRP) in sheep blood using a sliding-strip 3D µPAD. A) Selected images of the concentration-dependent response. B) Colorimetric response of the device as measured by the average color intensity. Here, the image was inverted and the Red, Green, and Blue (RGB) color values of the test zone were measured using ImageJ (in a circle with a diameter of 25 pixels at the center of the test zone). The RGB values of the control zone were subtracted from the RGB values of the test zone for each concentration of CRP, and then the R, G, and B values were averaged together to obtain the average color intensity. Some data points have been offset while plotting for clarity. C) The scatter plot represents mean±S.D. (n = 7–8 from data pooled over two experiments (where each experiment had 3–4 replicates) and hence pooled standard deviations were calculated), black dashed line represents a standard deviation-weighted linear fit to the mean, horizontal red line represents the detection limit (calculated as mean response of the 1 ng/mL sample + 3×S.D. of 1 ng/mL sample). D) Estimated receiver operating characteristic (ROC) curves for two “elevated” concentrations (10 and 30 ng/mL cut-offs) of CRP. These curves were obtained by varying the average color intensity cut-off from 5000 to 45,000 AU in increments of 1000 AU and calculating the number of true positives and true negatives at either 10 ng/mL or 30 ng/mL cut-off. The dotted line represents the expected ROC curve if the outcome from a diagnostic test was randomly guessed. The areas under the curve (a measure of accuracy) are approximately 0.89 and 0.83 for the cut-offs of 10 and 30 ng/mL respectively. (For interpretation of the references to color in this figure legend, the reader is referred to the web version of this article.)

Once the images were collected from the color scanner, they were inverted and the RGB color values of a circle with a diameter of 25 pixels in the center of the test zone in the sensing area were measured (similar to an approach we have described previously (Christodouleas et al., 2015)). The color intensity from each of the R, G, and B channels was averaged to obtain the average intensity. The average color intensity of the control zone was used as background and subtracted from the value of the test zone. All the measurements were performed using National Institutes of Health (NIH) ImageJ. The data presented in Fig. 2 are from two different experiments (each experiment included n = 3–4 devices for each concentration tested) and thus, pooled standard deviations (for each concentration) were used as an estimate of the standard deviation of the mean from the two experiments.

The receiver operator characteristics (ROC) curve was obtained by varying the cut-off for average color intensity from 5000 to 45,000 AU in increments of 1000 AU and then counting the true positive and true negative results to obtain the specificity and sensitivity, for each cut-off of 10 ng/mL and 30 ng/mL. The figures were plotted using OriginLabs Origin®.

## 3 Results and discussion

### 3.1 Design and operation of the sliding-strip 3D µPAD

The sliding-strip 3D µPAD comprises two principal parts: a sliding strip (fabricated in nitrocellulose, chromatography paper, and PET film) and a functional “dock”—a structure that surrounds a channel for the sliding strip, and that also distributes fluids (Fig. 1). The sliding strip contains the sensing area (which is a piece of wax-patterned nitrocellulose containing two zones: one is a control zone without capture antibody, and the other is a test zone with immobilized capture antibody) attached to a piece of wax-patterned chromatography paper (which is attached to a poly(ethylene terephthalate) (PET) film (transparency), to make handling more convenient, and to smooth the sliding of the strip). The sliding strip is labeled with the numbers 1, 2, and 3, and three alignment marks to guide the user during the three steps of operation of the device, and to ensure that the sensing area is aligned with the reagent areas of the functional dock (Fig. 1A). The sensing area has yellow wax circles (Fig. S4) around the active zone to provide better contrast than the black surroundings (black was used as the color for printing because preliminary experiments demonstrated that black wax, when printed, provides a better hydrophobic barrier than cyan, magenta, and yellow waxes). The functional dock comprises multiple layers of wax-patterned chromatography paper that are attached together with laser-cut double-sided adhesive tape. A layer of thick (2.45 mm) blotting paper located at the bottom of the device serves to provide a capillarity-driven flow of liquids used for washing the test zones, and to collect excess liquid and reagents (Fig. 1B). The top three layers (Fig. 1Bi–iii) split the added liquid (either sample or water) into two aliquots—one each for the control zone and the test zone in the sensing area. The docking layer (Fig. 1Biv) accommodates the sliding strip, while the isolation layer (Fig. 1Bv) completes the fluidic path and connects the sensing area of the sliding strip to the blotting paper.

To operate the sliding-strip 3D µPAD, we first ensure that the strip is inserted into the functional dock and aligned to position 1 (i.e. the number 1 from the sliding strip is visible and the red line marked at the bottom of the functional dock aligns with the red line above the number 1). We then add our sample (100 µL of whole blood, diluted 1:1000 with 1% w/v BSA in PBS) to the first inlet zone using a micropipette (or a syringe for resource-limited settings) and wash off excess sample with water (which dissolves and elutes the phosphate buffer salts stored in dry form in the paper) using a micropipette (150 µL or two drops when using a dropper for resource-limited settings) (Fig. 1C, Di). The next step is to move the sliding strip to position 2 by pulling the strip, to add more water (using a micropipette (150 µL) or dropper (two drops)) to the inlet to elute the stored detection antibody and stored buffer onto the sensing layer, and to wash off excess antibody with water (Fig. 1C, Dii). We perform the final step of ELISA by pulling the sliding strip to position 3, eluting the substrate (for the enzyme conjugated to the detection antibody) and stored detection buffer with water, and washing off excess unreacted substrate (Fig. 1C, Diii). Ultimately, we pull the strip out of the functional dock (Fig. 1C, Div), wait for half an hour for color development, and analyze the results visually or using a desktop scanner, based on the intensity of the color in the test and control zones.

### 3.2 Detection of C-reactive protein (CRP)

We chose CRP (a biomarker for sepsis (Benitz et al., 1998, Franz et al., 1999, Pierrakos and Vincent, 2010, Povoa et al., 2005), cardiovascular diseases (Ridker, 2003), inflammatory diseases (Hemila et al., 1987, Kahn et al., 1991, Lehtinen et al., 1986, Menees et al., 2015), and arthritis (Kim et al., 2015; Spoorenberg et al., 1999)) as the analyte of interest to test the ability of the sliding-strip 3D µPAD to perform ELISA. We consider three specific examples where the concentration of CRP can be used as a screening tool, when combined with other biomarkers, signs, or symptoms—neonatal sepsis, pelvic inflammatory disease, and inflammatory bowel diseases (IBD).

Serial measurements of CRP (if<10 µg/mL) in neonates can indicate that bacterial infection is unlikely (Benitz et al., 1998) and the sensitivity of this assay can be increased (from 93% to 96%) if the CRP levels are combined with measurement of interleukin-8 (IL-8) (Franz et al., 1999). This method of screening leads to a reduction of unnecessary antibiotic therapy in neonates in a cost-effective manner (Franz et al., 1999).

Elevated levels of CRP (>10 µg/mL (Hemila et al., 1987)) can be used as an indicator in women suspected of pelvic inflammatory disease (i.e. those that display symptoms such as adnexal tenderness and cervical motion tenderness (Kahn et al., 1991)). In addition, CRP levels can also be used to monitor therapeutic response of antibiotics in the case of neonatal sepsis and for pelvic inflammatory disease: CRP has a short half-life (19 h (Vermeire et al., 2006)) in blood and thus, the levels become normal quickly (as compared to other measurements such as erythrocyte sedimentation rates (Hemila et al., 1987)) in the case of effective therapy.

The levels of CRP in IBD can vary considerably (10–50 µg/mL for mild to moderate, 50–80 µg/mL for moderate to severe, and>80 µg/mL for severe disease (Vermeire et al., 2006)) and correlate better with Crohn's disease than with ulcerative colitis (Vermeire et al., 2006). A meta-analysis of published studies suggests that CRP levels<5 µg/mL could be used to exclude IBD in patients suspected of suffering from irritable bowel syndrome, while CRP levels>27 µg/mL suggest a 90% probability of IBD (Menees et al., 2015). Thus, we will look at two different cut-offs of ~ 10 µg/mL (for neonatal sepsis and pelvic inflammatory disease) and ~ 30 µg/mL (for IBD) to characterize the responses from our sliding-strip 3D µPAD.

We use a sandwich ELISA for the detection of CRP for two reasons: i) it has high specificity (since two antibodies are used and both bind to the analyte) and thus the potential for low false positive rates, ii) it is suitable for complex mixtures, since the analyte does not need to be purified before use (as we demonstrate here in our simulated CRP-elevated blood). Since the levels of CRP can increase 1000-fold compared to physiological levels during inflammation (Schmit and Vincent, 2008), there is a possibility of hook effect (i.e. a high concentration of CRP saturates the detection and capture antibodies, prevents the complex of detection and capture antibodies from forming, and hence reduces the response) but the washing steps in our assay (and physical separation of capture and detection zones) should prevent unbound CRP from contacting the detection antibody and hence minimize this concern.

The components of the CRP ELISA are commercially available, although typically the detection antibody is used as a conjugate to horseradish peroxidase, and 3,3′,5,5′-tetramentylbenzidine (TMB) is used as the colorimetric molecule (in the presence of hydrogen peroxide, which is unstable and difficult to store in a paper device). We modified the procedure to use alkaline phosphatase (ALP) as the enzyme linked to the antibody, and BCIP/NBT as the colorimetric indicators (or substrate), because this substrate is available in a tablet form (which can be crushed to a powder) and thus, can be stored between two layers of paper. We conjugated ALP-streptavidin to the biotinylated detection antibody by mixing the two solutions at room temperature for 15 min. We spotted this ALP-detection antibody mixture on the second row of the functional dock in the storage layer and dried it at 37 °C (~ 15% relative humidity (RH)) for 30 min in ambient air (see Supplementary information for details on quantities). We placed the substrate between the inlet layer and the splitting layer (Fig. 1B) in powder form using a custom-built template. The capture antibodies were spotted on the test zone (the right hole of the sensing area on the sliding strip) and then the sample and control zones were blocked (i.e., treated to inhibit non-specific adsorption of proteins) using a solution of 3% w/v BSA in PBS.

Our sliding-strip 3D µPAD can detect CRP with some accuracy at concentrations in the range of 1–100 ng/mL (Fig. 2). Thus, to measure the clinically relevant concentrations of CRP in blood (~ 1–10 µg/mL in “normal” patients and “elevated” levels of ~ 100 µg/mL in inflammatory conditions), the sample would have to be diluted 1000-fold before measurement. We accomplish this dilution by filling blood in a fixed volume (1 µL) capillary and then shaking the capillary in a tube containing a fixed volume of diluent (1 mL 1% w/v BSA in PBS).

**Fig. 3:**
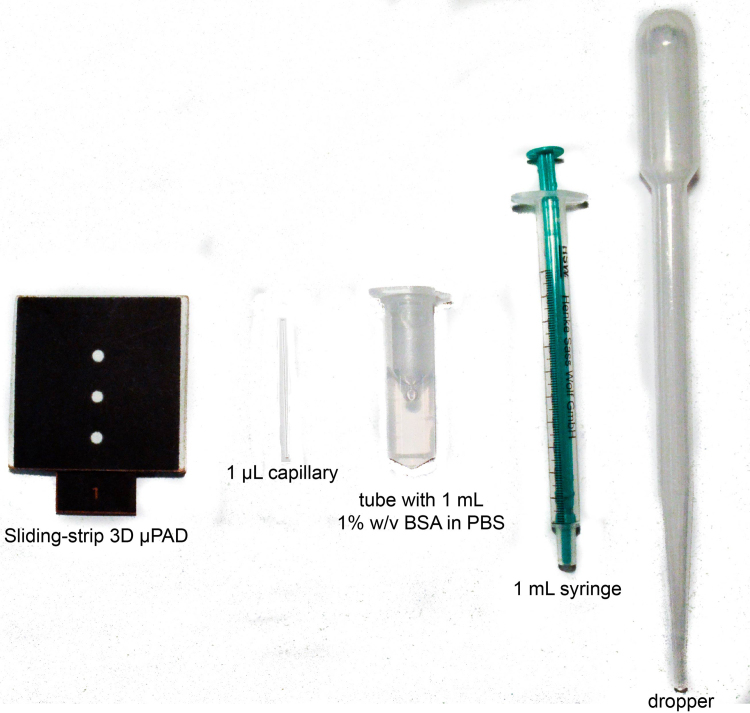
Components of a kit to be used with sliding-strip 3D µPAD in resource-limited settings: the sliding strip and the functional dock, assembled together; a 1 µL capillary to collect blood; a tube with pre-filled 1 mL of 1% w/v BSA in PBS (to be used as diluent for obtaining 1000-fold dilution of blood); a 1 mL syringe to measure out 0.1 mL of the sample; a plastic dropper to add 2 drops of water for each of the three inlets.

**Fig. 4:**
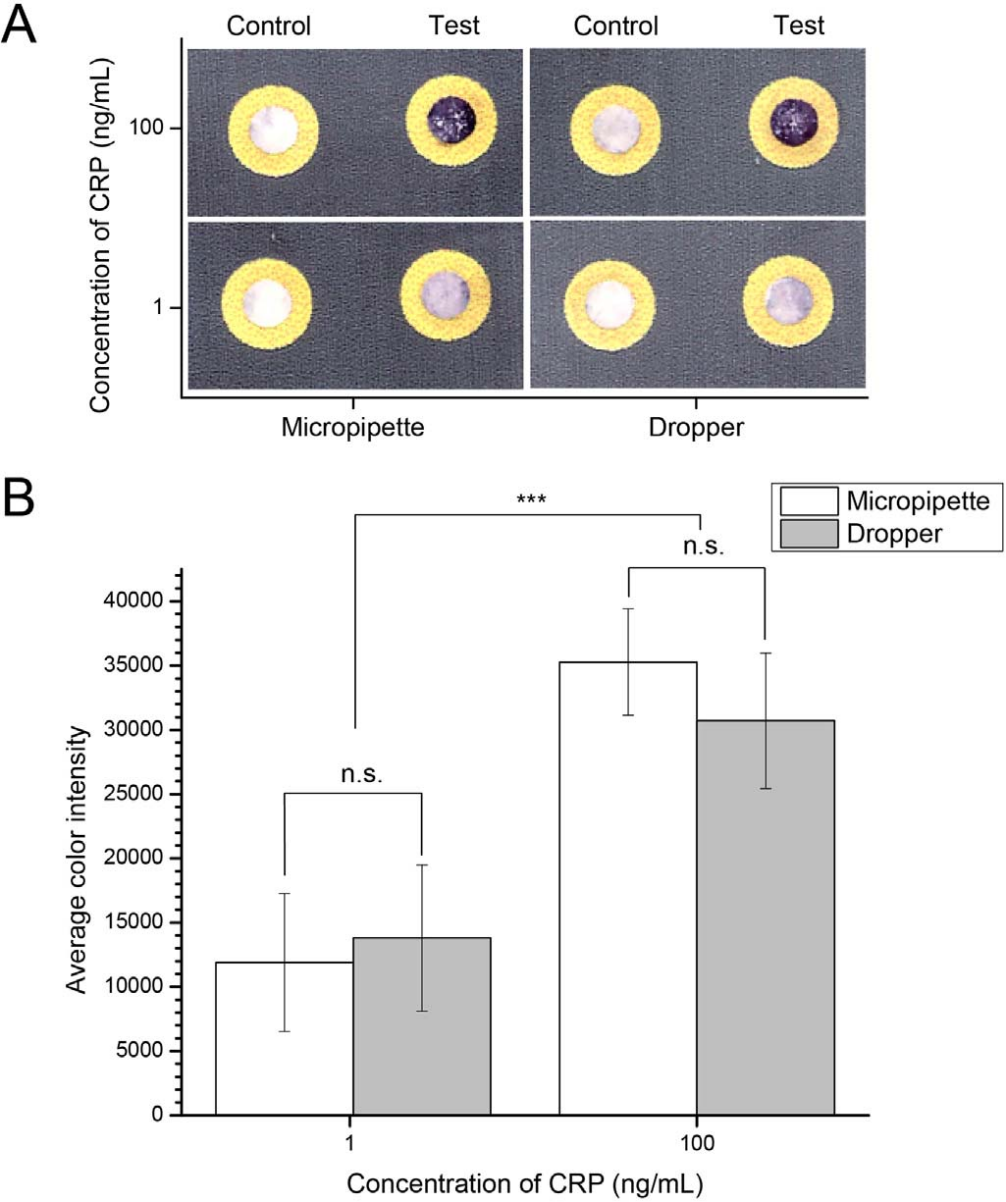
Comparison of the use of a micropipette and a dropper for assaying C-reactive protein (CRP) using a sliding-strip 3D µPAD. A) Representative images of the results. B) Response of the device as measured by the average color intensity. Average color intensity was calculated in the same way as in Fig. 2. The column plot represents mean±S.D. (n = 6), n.s.: not significant (p>0.1), *** (p<0.001).

A disposable plastic syringe can be used to add the sample (1000-fold diluted blood) to the first inlet of the device, and a plastic dropper can be used to add water to the second and third inlets for the washing and eluting steps (Fig. 3 shows the components of a kit that could be deployed in resource-limited settings). Using this kit, we compared the results of experiments conducted using a micropipette and a dropper in Fig. 4, and showed that there is no significant difference between these two methods of executing steps in the assay. Both methods result in a significant (p<0.001) difference in the average color intensity for the “normal” and “elevated” levels of CRP (1 ng/mL and 100 ng/mL in diluted blood samples respectively). This level of statistical significance, by itself, is useful in characterizing the assay, and particularly in its potential for future development: it is not very useful in suggesting how this test—at this level of development—would be used in the clinic. We discuss this issue in the Conclusions section.

### 3.3 Accuracy

The accuracy of sliding-strip 3D µPAD can be estimated by looking at different cut-offs for CRP levels. Fig. 2 shows the results obtained for different concentrations of CRP (and Fig. S6 illustrates some statistical parameters—mean, median, and quartiles—in a box plot). The limit of detection (as calculated by using the linear fit in Fig. 2C and considering the mean response from 1 ng/mL sample + 3 × S.D. (standard deviation) of the 1 ng/mL sample) of the assay is 40 ng/mL (or ~ 350 pM) CRP (or 40 µg/mL in undiluted blood). While the mean values of the color intensity show a linear trend with increasing concentration (Fig. 2C and Fig. S7), further optimization of the sensing area will be necessary to quantify the concentration of CRP accurately in the range of 40–100 ng/mL.

In its current form, the sliding-strip 3D µPAD can be used as a pre-screening tool. Although the data presented in Fig. 2B are not complete for calculating ROC curves (because the data were obtained from replicates of spiked blood samples at various specific concentrations, rather than from blood samples drawn from a sample of a population of humans), the data can nonetheless be used to *estimate* ROC curves for various cut-offs and assess the expected performance of the sliding-strip 3D µPAD. At the cut-offs of 10 ng/mL (10 µg/mL in undiluted blood as a relevant value for neonatal sepsis and pelvic inflammatory disease) and 30 ng/mL (30 µg/mL in undiluted blood as a relevant value for IBD), the accuracies of our device are 89% and 83% (as estimated by the areas under the curves in Fig. 2D). In the case of neonatal sepsis and pelvic inflammatory disease, the consequence of an undetected infection (false negative) is more serious than that of a false positive. The expected sensitivity of detecting “elevated” CRP is about 76% for a specificity of 86%, which demonstrates moderate performance. (We would get the same estimate for accuracy if we wanted to consider the test for ruling out IBD, i.e. CRP cut-off<5 µg/mL, because of the resolution of our measured concentrations of CRP, which are at intervals of ~ 20 µg/mL of undiluted blood.) Similarly, when comparing IBD and irritable bowel syndrome, the consequences of misdiagnosing patients with IBD as irritable bowel syndrome (false negative) are more severe than the other way round (false positive) (Menees et al., 2015). So, when we look at a CRP cut-off of 30 ng/mL, a comparable sensitivity (to the ~ 10 ng/mL ROC curve) of about 80% is obtained at specificity of about 73% and thus, this method of screening can be used to indicate further investigation into other biomarkers such as fecal calprotectin (Menees et al., 2015).

### 3.4 Cost

We have estimated the bill of materials (BOM), for fabrication in small lots (300 devices) of a pouched kit in our research lab at $2.37 per test ($2.15 for materials and $0.21 for packaging), excluding labor and overhead (Tables S1 and S2). With advice from Diagnostics for All, Inc., we have also estimated that the cost of goods, *including* labor and overhead, and using the larger-batch manufacturing line at their facilities (2000 devices per week), would be at $4.29 per test (as opposed to $15.19 if produced in our lab at the rate of 300 devices per week). We estimate that the final cost of goods (including materials and labor overhead) could decrease 10-fold to ~ $0.50 if we transferred fabrication to a roll-to-roll manufacturing process, and manufactured at a volume of 100,000 devices/week. We can compare this *cost* to the *price* of $1.65 per test (based on commercially available ELISA kits DY1707 and DY008 from R&D Systems). The commercially available kits need to be executed in a fully equipped lab (with micropipettes, plate readers, and trained personnel) which adds an additional cost of operation.

### 3.5 Comparison to similar devices

The sliding-strip 3D µPAD described here, the SlipChip (Du et al., 2009, Li et al., 2010a, Liu et al., 2010, Ma et al., 2014, Shen et al., 2010a, Shen et al., 2010b, Shen et al., 2011), and the paper machine (described previously for analysis of *E. coli malB* gene (Connelly et al., 2015)) all rely on the relative movement of two pieces of material (i.e. paper/nitrocellulose/PET for the sliding-strip 3D µPAD, glass/plastic for the SlipChip, magnetic strip/laminate for the paper machine), with patterned channels, to make and break fluidic contacts/connections during an assay.

Our sliding-strip 3D µPAD would, we believe, ultimately (with greater accuracy) be more suitable for low-cost, point-of-care diagnostics than the SlipChip, for five reasons. (i) It is simpler to fabricate than the SlipChip. (ii) It can readily form 3D microfluidic channels (which are useful for distributing fluidic samples into densely arrayed test zones (Martinez et al., 2008b)) on the device by stacking layers of paper and adhesive tape; this type of fluidic distribution system is difficult to realize in the glass/plastic-based SlipChip. (iii) It can be easily disposed of by incineration. (iv) It has a lighter weight and smaller volume, and is more easily transported than the SlipChip; it is also relatively insensitive to mechanical damage or breakage. (v) It uses capillary action to transfer fluid whereas SlipChip requires the use of an external pumping mechanism. The sliding-strip 3D µPAD can be more accurate (89% accuracy for 10 ng/mL cut-off of CRP) compared to a platform based on the SlipChip (76% accuracy for a 50 ng/mL cut-off of myoglobin, a biomarker for myocardial infarction) (Song et al., 2016). An obvious shortcoming of the sliding-strip 3D µPAD compared to the SlipChip is that our device requires larger volumes (microliters) of sample and water than the SlipChip (nanoliters). In the current format, it is also less versatile in the range of assays it can conduct than the SlipChip (Begolo et al., 2013, Begolo et al., 2014, Du et al., 2009, Li et al., 2010a, Liu et al., 2010, Ma et al., 2014, Shen et al., 2010a, Shen et al., 2010b, Shen et al., 2011). While the limit of detection using SlipChip is reported to be better (~ 13 pM insulin, (Liu et al., 2010) with a potential for single molecule detection using digital immunoassays (Ge et al., 2014)) than our device (~ 350 pM CRP) for immunoassays, SlipChip utilizes fluorescence for its output, whereas the sliding-strip device uses color, and is therefore not expected to be as sensitive.

Six features differentiate our sliding-strip 3D µPAD from the device described by Connelly et al. (paper machine). (i) The paper machine executes molecular assays whereas our sliding-strip device performs immunoassays. The requirements for the two types of assays are quite different. (ii) The paper machine requires the user to add all reagents (wash buffers, master mix, SYBR Green I) in a liquid form. Our device stores all required reagents (buffers, antibodies, enzymatic substrate) within the device, and only requires the user to add sample and water. (iii) The paper machine uses magnetic strips to hold layers together whereas we use paper and tape. (iv) The paper machine uses paper only as discs in the active zones, whereas the sliding-strip device uses paper as the base for all its components. This feature makes the sliding-strip devices lighter in weight, and in principle less expensive, than the paper machine. (v) The magnetic rubber layers act as barriers between reaction zones to prevent cross-contamination by movement of fluids between sample and control zones in the paper machine; the sliding-strip device uses wax for controlling the flow of fluid and avoiding contamination. (vi) The paper machine requires that sample, negative control, and positive control be added separately to different zones. In our device, the splitting layer allows us to add fluids to a single inlet. This fluid is automatically split into control and test zones.

## 4 Conclusions

This paper extends the capabilities of 3D µPADs by introducing a new sliding-strip design (we have described older implementations of this concept previously (Connelly et al., 2015; Liu et al., 2011)) and now allows 3D µPADs to perform an assay at the level of complexity of an ELISA by a procedure that involves five steps: i) obtaining a sample of blood (by finger prick); ii) diluting the blood to a range useful for this assay (using the kit illustrated in Fig. 3); iii) adding the diluted blood, and water, to the first zone; iv) moving the sliding strip between zones and adding water to the respective zones, on a timed schedule; and v) reading the intensity of color (relative to a color bar) using a scanner (or by eye). The entire procedure would take approximately 90 min in practiced hands.

The sliding-strip 3D µPAD has six useful features as a diagnostic tool. (i) It provides an assembly that can be developed to perform ELISA in resource-limited settings. (ii) It could be inexpensive when produced on a large scale (estimated cost of goods is ~$0.50/kit in large volumes of ~ 100,000/week). (iii) It provides colorimetric results and thus, is compatible with telemedicine (Martinez et al., 2008a) based on cell-phones, where the results can be quantified on-site via a portable imaging device (e.g., camera phone or portable scanner) and then transferred off-site for further analysis (Chin et al., 2013, Estrin and Sim, 2010, Lee et al., 2011, Lillehoj et al., 2013, Mudanyali et al., 2012, Nemiroski et al., 2014, Zhang and Liu, 2016). (iv) Its operation requires some, but relatively limited, training and experience. (v) All the reagents (buffers, antibodies, enzyme, and substrate) are stored in the device and the operator only needs to add sample and water. (vi) It has the potential to be adapted for different types of biochemical assays (e.g. sandwich, direct, or indirect ELISAs (Lequin, 2005)) by changing the stored reagents.

Conducting ELISA in a portable format has four advantages over conventional 96-well plate ELISA. (i) It uses simpler equipment (i.e. a fixed-volume capillary tube, a disposable plastic syringe, a plastic dropper, and a portable imaging device). (ii) It does not require the operator to handle buffers, biomolecules, or colorimetric substrates (only ~ 450 µL of water is needed to run one assay). (iii) It demands limited skill from the operator to perform the assay, and thus has the potential to permit point of care diagnostics with minimal training. (iv) It is useful in point of care tests for single patients or low-volume clinics where 96-well plates would be inappropriate. One of the drawbacks of the sliding-strip 3D µPAD is that the detection limit (40 ng/mL in diluted blood) of CRP compared to the 96-well format (~ 20 pg/mL (Krause et al., 2015)) is worse by approximately 10^3^; nonetheless, the sliding-strip device can detect CRP in the clinically relevant concentration range (~ 1–100 µg/mL). The sensitivity of the sliding-strip device could be improved by using a substrate such as fluorescein diphosphate (Liu et al., 2010) for enzymatic conversion such that the product of enzymatic amplification is ultimately detected by fluorescence.

The major deficiency of the sliding-strip 3D µPAD, at its current stage of development, is its limited accuracy (best summarized in Fig. 2, S6, and S7). The current metric for the limit of detection is 3× standard deviation (S.D.) of the blank (1 ng/mL CRP) sample + the value of the blank sample. The S.D. shown in Fig. 2C suggests a limit of detection ≥ 24,000 AU. In fact, because the focus of this work was on demonstrating the construction and the operation of the device, we have only measured a limited number of samples (n = 7–8) to estimate an S.D., a more accurate value would require n ~ 30. The reasonable fit (R^2^ = 0.96) of the mean values to a line relative to the concentration of CRP suggests, however, that the large value of S.D. does reflect uncontrolled random errors (rather than systematic errors) in the construction of the device, and that attention to detail would reduce them. Plausible candidates for improvement are measuring out powdered reagents, non-uniformity/masking of color, and misalignment of the test zone on the sliding strip and the fluidic zones in the dock.

Even at this level of uncertainty, however, this test would be useful (in conjunction with history of symptoms, clinical signs, and other biomarkers) in pre-screening, when the time required and/or logistics involved to reach a gold-standard assay in a central laboratory are prohibitive (for example, in a rural clinic). The cut-off would depend on the application being considered (e.g. an expected value of ~ 17,000 AU for 10 µg/mL in undiluted blood for screening of neonatal sepsis or pelvic inflammatory disease). For example, a value higher than 17,000 AU would be considered abnormal and a value of about 40,000 AU would be clear evidence of urgent diagnosis for neonatal sepsis. No analysis from the test would be considered diagnostic, but even at its current state of development—since it could be made available in villages in clinics removed from central laboratories—it could justify the time and effort needed to transport patients (or samples) to a central laboratory, and/or to assemble a package of data (e.g. CRP from this test, troponin from a similar ELISA, electrocardiogram (ECG) from a cellphone-based reader, patient symptoms, and medical history) for referral to a higher-level healthcare team.
