# Supplementary material for: Sliding-strip microfluidic device enables ELISA on paper
Source: Biosens Bioelectron. 2018 Jan 15;99:77–84. doi: 10.1016/j.bios.2017.07.034 (PMC5628584; doi:10.1016/j.bios.2017.07.034)
Supplement: Supplementary file 1 — Supplementary material [file mmc1.pdf]

# Supporting information

**for**

# Sliding-strip microfluidic device enables ELISA on paper

Mohit S. Verma[1], Maria-Nefeli Tsaloglou[1], Tyler Sisley[1], Dionysios Christodouleas[1], Austin Chen[1], Jonathan Milette[1], and George M. Whitesides[1, 2, 3 *]

[1]Department of Chemistry and Chemical Biology, Harvard University, 12 Oxford Street, Cambridge, MA 02138, USA.

[2]Wyss Institute for Biologically Inspired Engineering, Harvard University, 60 Oxford Street, Cambridge, MA 02138, USA.

[3]Kavli Institute for Bionano Science and Technology, Harvard University, 29 Oxford Street, Cambridge, MA 02138, USA.

## Materials and Methods

*Materials and equipment*

Whatman$^{TM}$ grade 1 chromatography paper, Teknova 10x detection buffer solution (1M Tris-HCl, 1M NaCl, pH 9.5), and defibrinated sheep blood (10052-776) were purchased from VWR (Radnor, PA, USA). Nitrocellulose membrane, 0.45 µm and extra thick (2.45 mm) blotting filter paper were purchased from Bio-Rad Laboratories (Los Angeles, CA, USA). Double-sided adhesive (FLX000546) was purchased from FLEXcon (Spencer, MA, USA). Anti-human C-reactive protein (CRP) capture antibodies and biotinylated anti-human CRP detection antibodies were purchased as a part of an R&D CRP ELISA kit (DY1707) from Fisher Scientific (Boston, MA, USA). Recombinant human CRP (1707CR200) and Tween$^{TM}$ 20 were also purchased from Fisher Scientific. Streptavidin-alkaline phosphatase (ALP) was purchased from Life Technologies (Chicago, IL, USA). 5-bromo-4-chloro-3-indolyl phosphate (BCIP)/nitro blue tetrazolium (NBT) tablets, 10% w/v bovine serum albumin (BSA) in phosphate buffered saline (PBS, pH 7.4), and 10x PBS (pH 7.4) were purchased from Sigma-Aldrich (Atlanta, GA, USA). All reagents were used without further purification. A Xerox ColorQube 8870 solid ink printer was used to pattern wax on nitrocellulose membrane and chromatography paper. A VersaLASER® VLS 3.50 laser cutter was used to cut patterns in layers of double-sided tape and in the docking layer. A Stratasys Fortus 250 mc 3D printer was used to print an acrylonitrile butadiene styrene (ABS) template for aligning all the paper and tape layers. Water obtained from a Millipore purification system (resistivity > 18 MΩ.cm) was used throughout.

*Fabrication*

Wax patterns were first printed on chromatography paper to form layers of the functional dock (Figures S1, S2, S3, and S5) and then the paper was heated using a heat gun (Steinel 3481) to melt the wax and allow it to penetrate into the paper matrix. Similarly, wax patterns were printed on nitrocellulose membranes (Figure S4) to form the sensing layer and the wax was melted by placing the membrane in an oven at 140 °C for one minute. Three-hole or six-hole patterns were then cut on double-sided adhesive using the Versa laser cutter. These tape layers were used in assembling the 3D µPAD as shown in Figure 1. During assembly, 5 µL of a solution containing detection antibody (prepared by mixing three solutions: 0.5 µL of 2 mg/mL ALP-streptavidin, 49.5 µL of 1% w/v BSA in PBS, and 50 µL of 16.2 µg/mL biotinylated anti-human CRP antibody) was added to each of the holes in the second row of the storage layer and this layer of chromatography paper was allowed to dry at 37 °C for 30 minutes. On the inlet layer, 10 µL of 10x PBS with 0.25% Tween$^{TM}$ 20 was spotted onto zone 1 and zone 2, while 10 µL of 10x detection buffer was spotted onto zone 3. The inlet layer was allowed to dry for 37 °C for one hour. A template made up of tape-paper-tape sandwich (while leaving the tape backing on, to prevent it from sticking to the device) with laser-cut holes was used to control the amount of BCIP/NBT powder (formed by crushing the commercial tablets using mortar and pestle) deposited in the splitting layer. The powder was first filled in the holes and packed with a plastic spatula, then the tape-paper-tape template was removed to leave a fixed amount of powder behind. (This method of depositing powder was used because it was rapid and inexpensive; its repeatability probably requires improvement.) The splitting layer was covered by the adhesive inlet layer to seal the substrate in place. Then, the inlet/splitting layers were added to the storage, docking (which comprises two sheets of chromatography paper attached together using double-

sided tape and laser-cut to accommodate the sliding-strip), and isolation layers by stacking and using double-sided adhesives between each chromatography paper. The entire stack was then attached to blotting paper using double-sided adhesive as well. The sensing area was fabricated by adding 2 µL of a solution of capture antibody (360 µg/mL in PBS containing 1% w/v BSA) to the test zone (a circle of 2 mm diameter located on the right side of the sensing area) of the wax-patterned nitrocellulose membrane and allowing it to dry at 37 °C for 30 minutes. The nitrocellulose membrane was washed three times in PBS with 0.05% Tween™ 20 to remove excess antibodies. The membranes were placed in a petri dish filled with 3% w/v BSA in PBS and incubated on an orbital shaker for one hour. The membranes were washed once with PBS and twice with Millipore water to remove excess reagents and dried at 37 °C (~15% relative humidity (RH), ambient pressure) for 30 minutes. The nitrocellulose strips were attached to the sliding-strip pattern on chromatography paper on the top section using double-sided adhesive. Finally, a poly(ethylene terephthalate) (PET) film (transparency) was attached to the sliding strip on top of the chromatography paper (using double-sided tape) to make handling of the strips more convenient. The sliding strip was placed in the functional dock and the assembly was pressed using a mechanical press (15” x 15” Power Heat Press, model OX-A1) at room temperature to provide conformal contact between all the layers of the sliding-strip 3D µPAD. The markings on the sliding strip were aligned to the bottom marking of the functional dock to ensure that the sensing area is in the correct location, before running the assay.

**Supporting Figures**

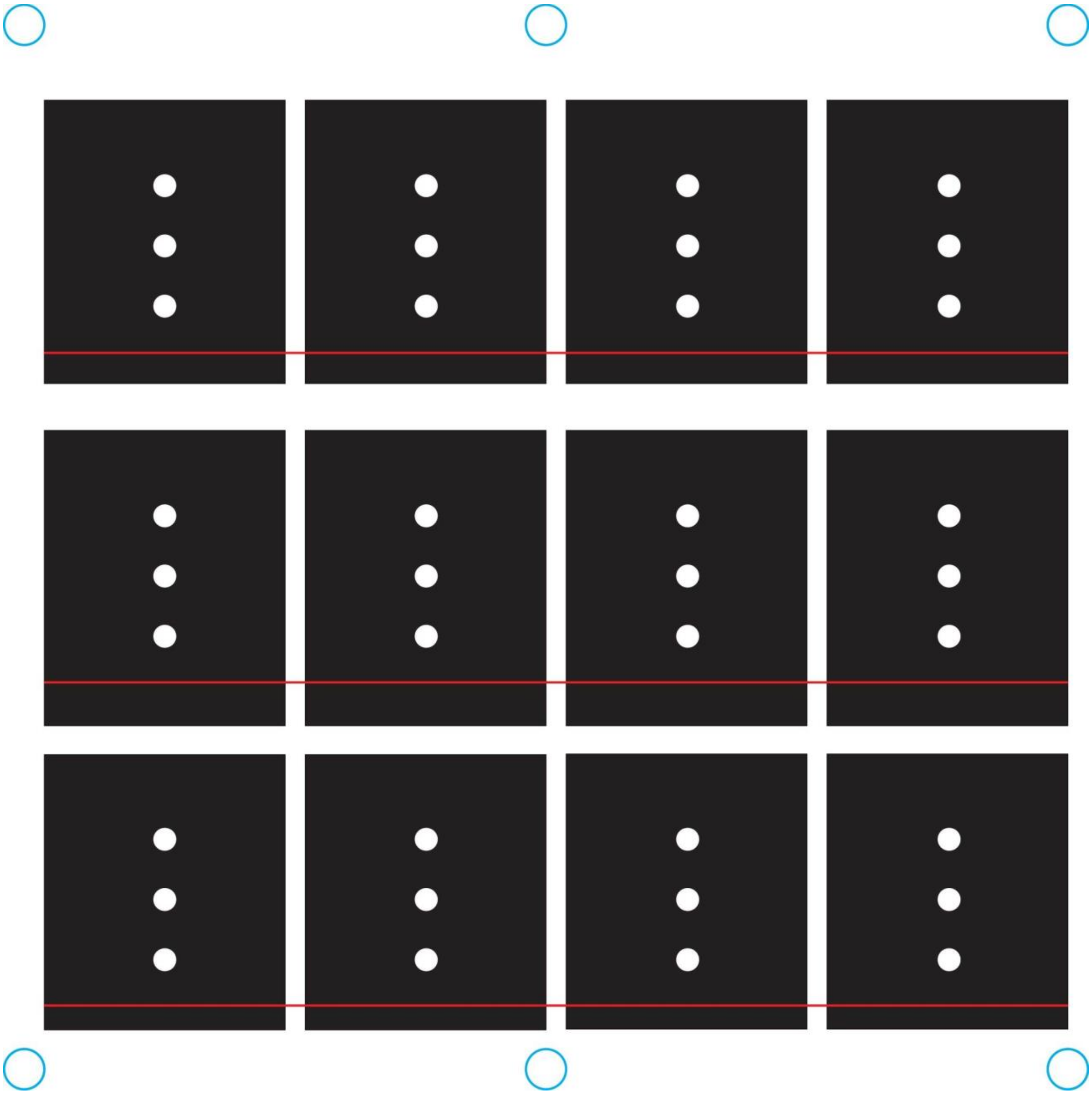

**Figure S 1:** Wax layout for inlet layer of the functional dock (to be printed on chromatography paper).

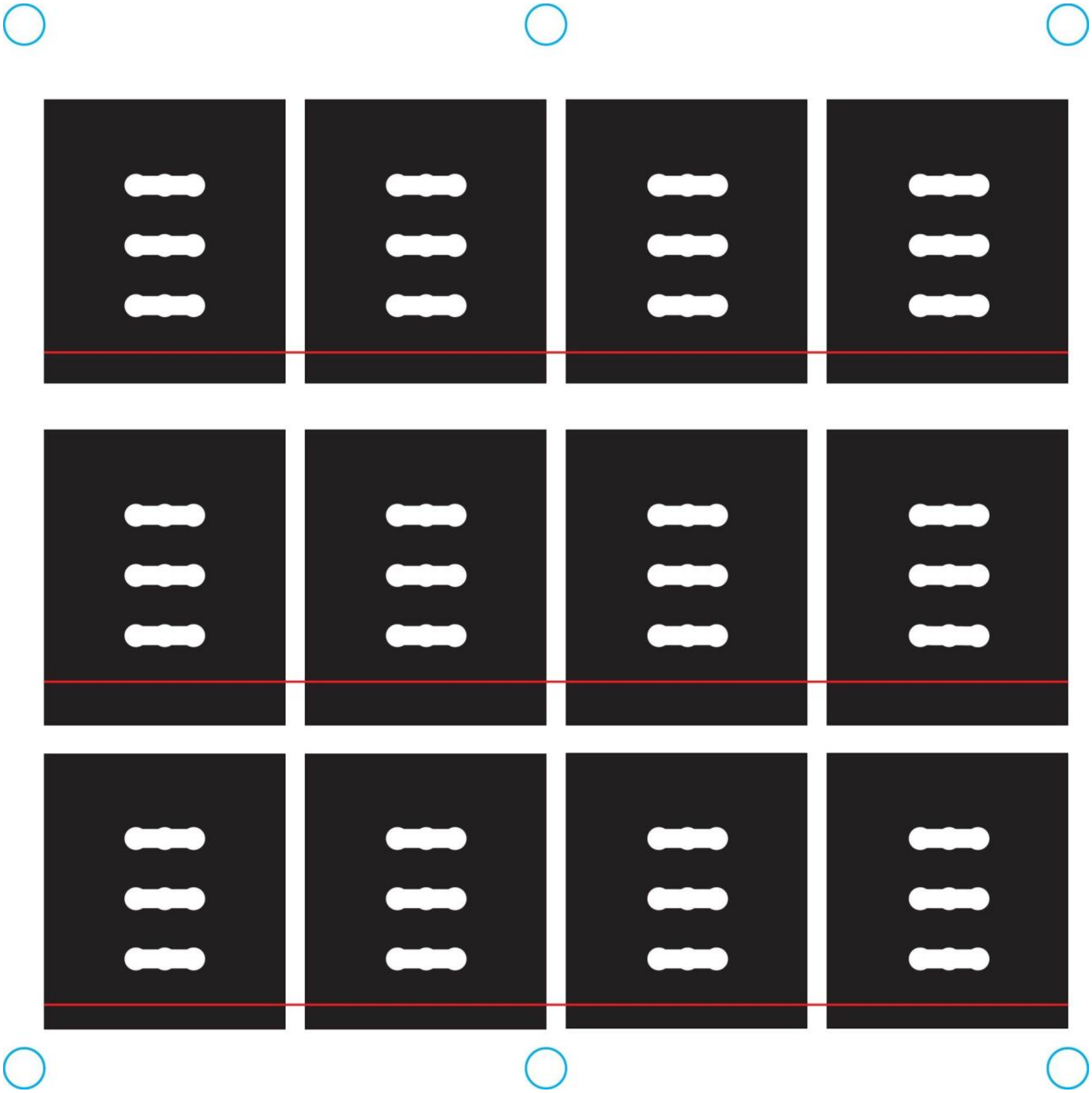

**Figure S 2:** Wax layout for splitting layer of the functional dock (to be printed on chromatography paper).

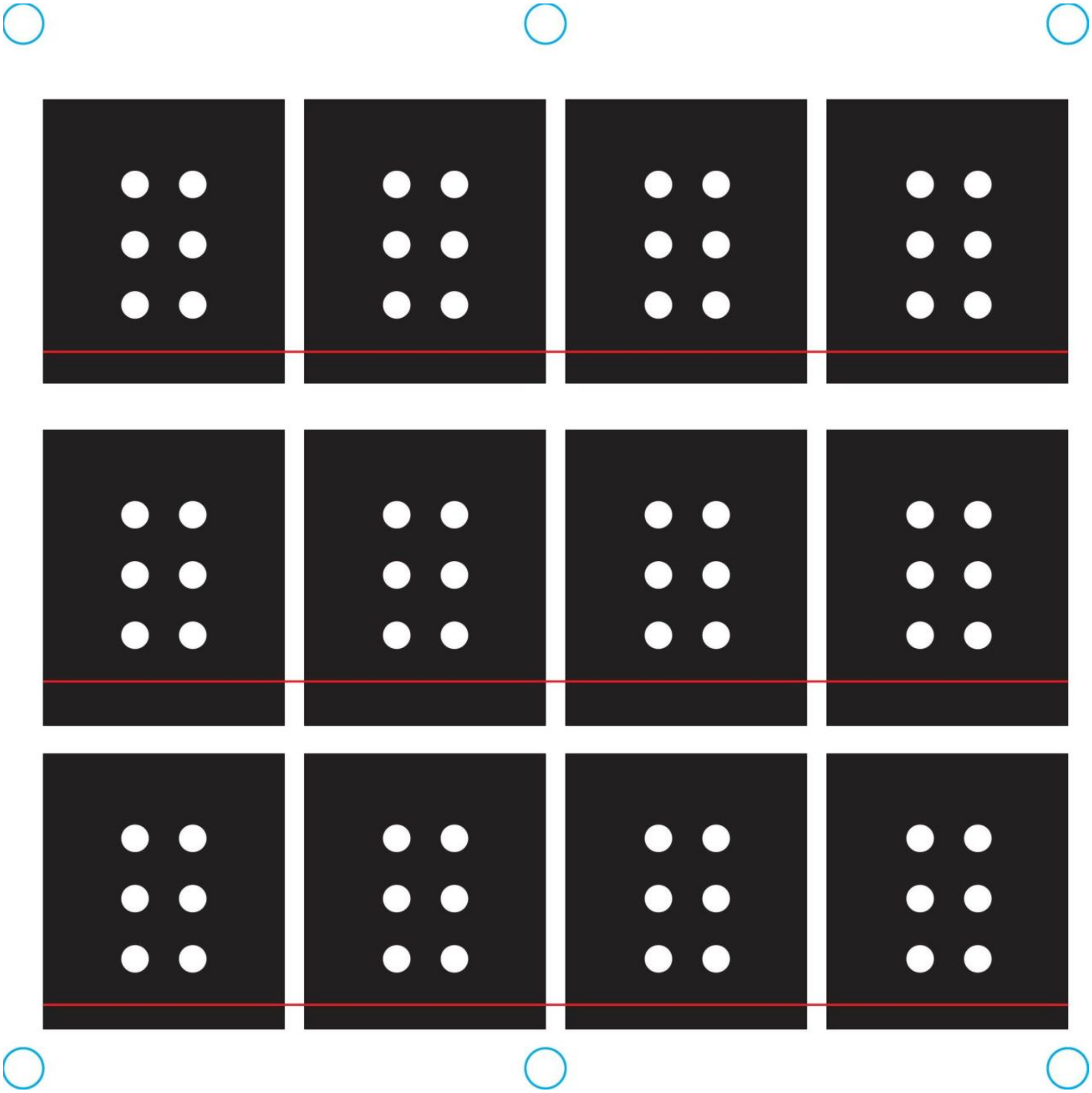

**Figure S 3:** Wax layout for storage and isolation layers of the functional dock (to be printed on chromatography paper).

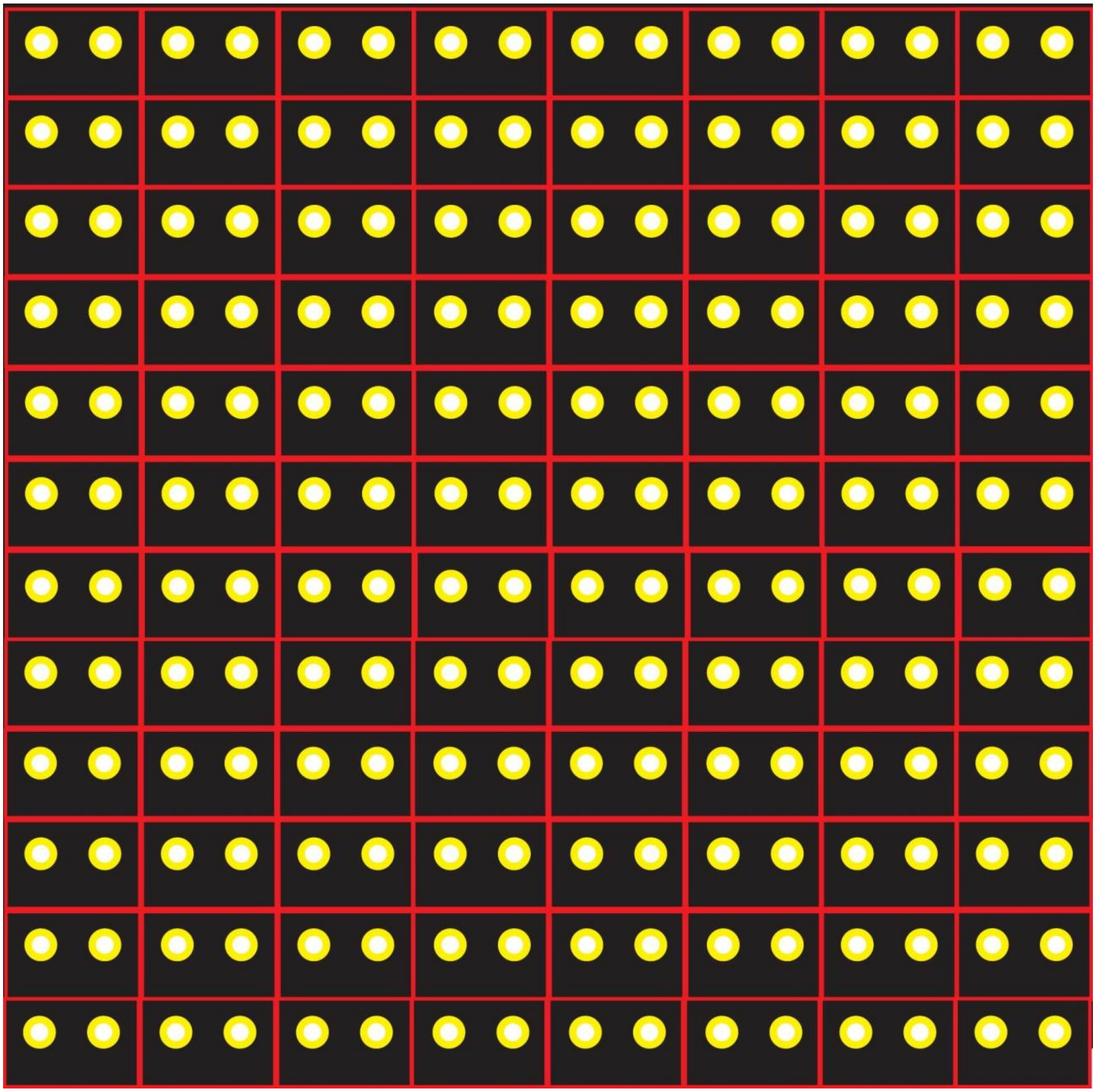

**Figure S 4:** Wax layout for sensing area (to be printed on nitrocellulose) of the sliding strip. The yellow circles are used around the white sensing area to provide a better contrast of the colorimetric output (purple-blue) compared to the black surrounding area.

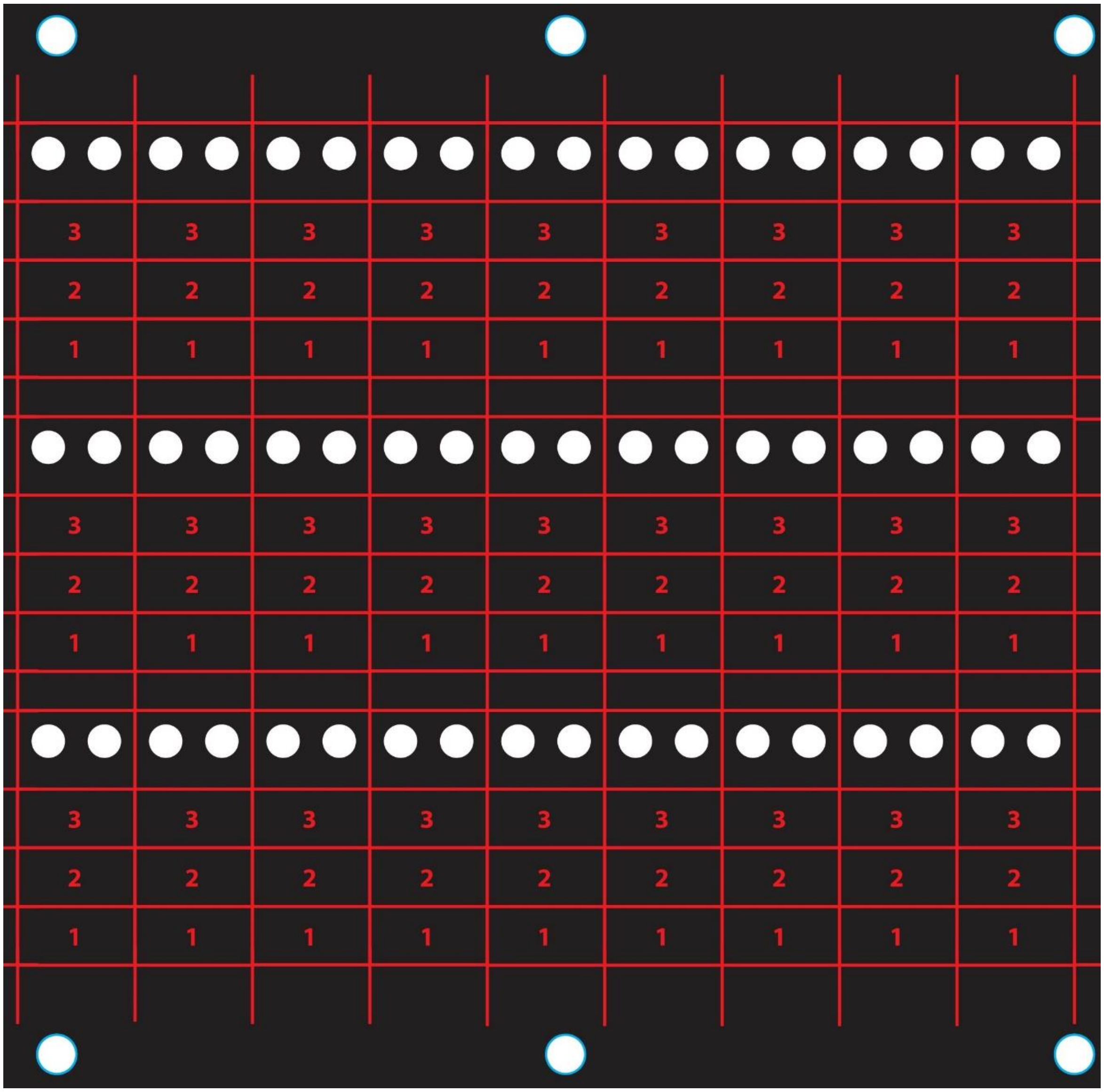


**Figure S 5:** Wax layout for the sliding strip backbone (to be printed on chromatography paper).

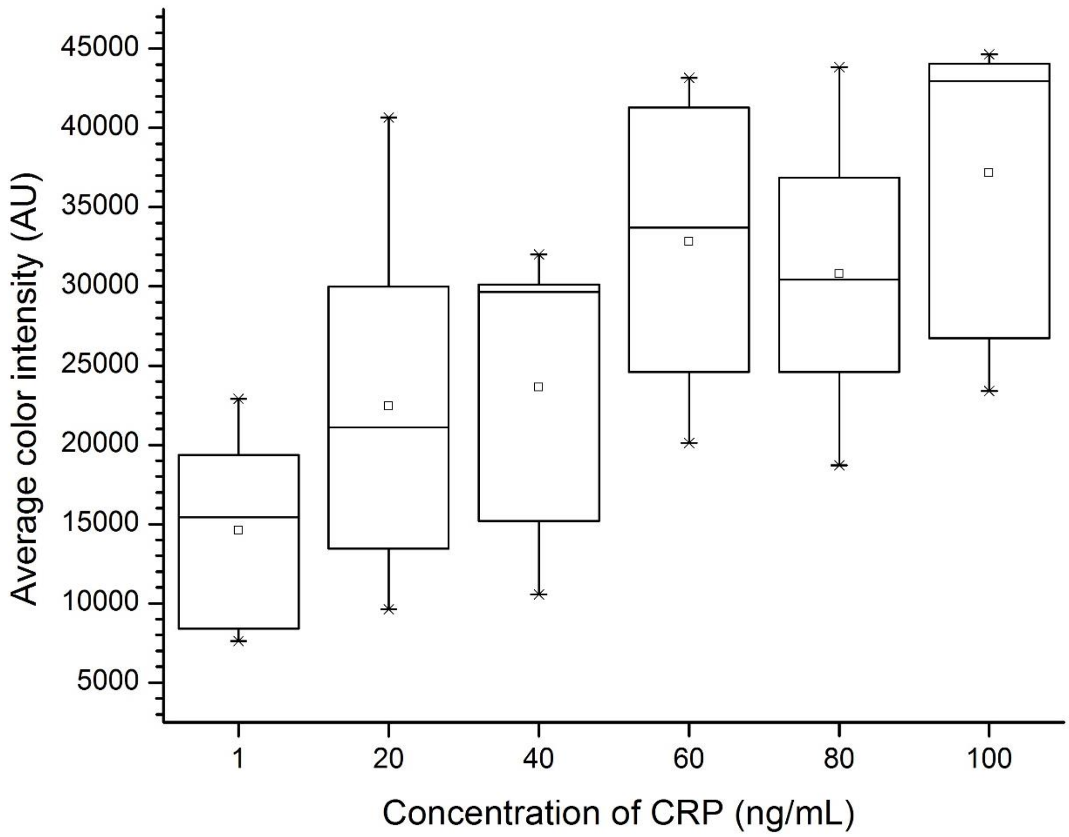


**Figure S 6:** Box plot of the colorimetric response (scatter plot is shown in Figure 2) from sliding-strip 3D µPAD when detecting various concentrations of CRP spiked in sheep blood (n = 7-8).

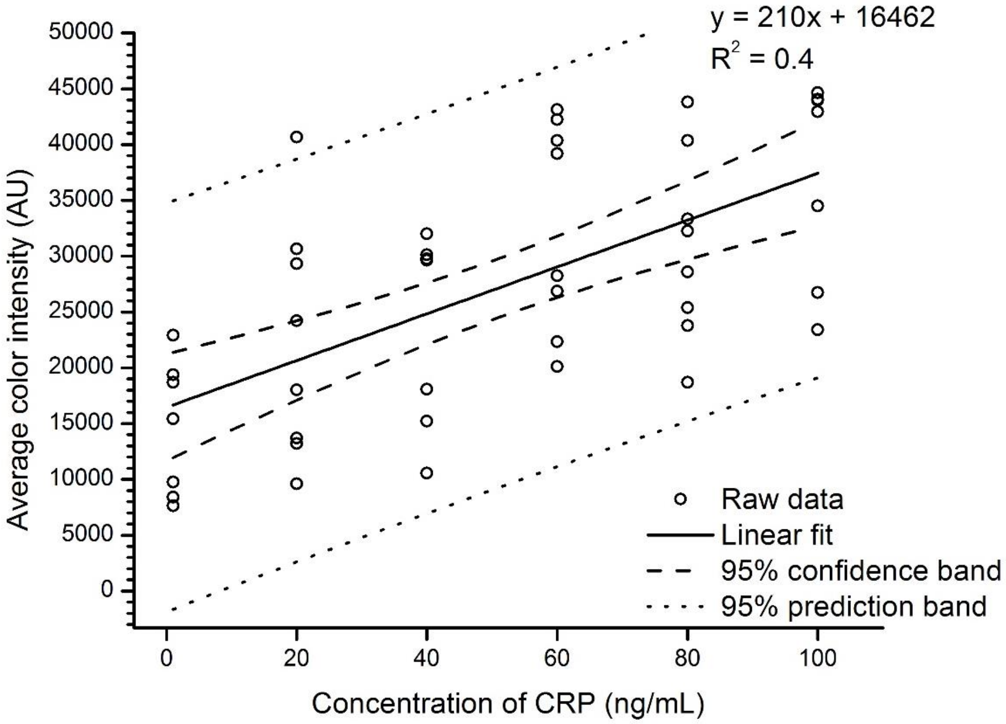


**Figure S 7:** Scatter plot of the colorimetric response of sliding-strip 3D µPAD along with the linear fit and the 95% confidence band and 95% prediction band (calculated using OriginLab Origin ® linear regression). The 95% confidence band estimates the confidence intervals for the fitted line such that a line of best fit of means would fall within the confidence band 95% of the time. The 95% prediction band estimates the region where 95% of the individual data points are expected to fall in future measurements.

**Table S1:** Bill of materials of a kit for sliding-strip 3D µPAD (sorted by % contribution). Listed prices are those that a typical research laboratory would pay for the products.

| Item | Supplier | Catalog # | Quantity | Units | Price (USD) | Unit price | Scrap rate | Corrected Unit Price | # of units/kit | Contribution to cost/kit | % Contribution to total cost |
|---|---|---|---|---|---|---|---|---|---|---|---|
| Detection antibody | Fisher Scientific | 842677 | 1 | mL | 200 | 100.0000 | 0.1000 | 110.0000 | 0.0050 | 0.5500 | 26% |
| NBT/BCIP tablets | Sigma-Aldrich | 11697471001 | 20 | tablet | 91 | 4.5500 | 0.1000 | 5.0050 | 0.0833 | 0.4171 | 19% |
| Grade 1 Chromatography paper 20 x 20 cm | VWR | 21427-003 | 100 | sheet | 49.16 | 0.4916 | 0.1000 | 0.5408 | 0.5370 | 0.2904 | 13% |
| Capture antibody | Fisher Scientific | 842676 | 1 | mL | 200 | 100.0000 | 0.1000 | 110.0000 | 0.0020 | 0.2200 | 10% |
| Extra Thick Blot Filter Paper, Precut, 19 x 18.5 cm | Bio-Rad | 1703969 | 30 | sheet | 51.85 | 1.7283 | 0.1000 | 1.9012 | 0.0833 | 0.1584 | 7% |
| Nitrocellulose Membrane, Precut, 0.45 µm, 15 x 15 cm | Bio-Rad | 1620116 | 10 | sheet | 114.75 | 11.4750 | 0.1000 | 12.6225 | 0.0104 | 0.1315 | 6% |
| 1 mL Syringe | VWR | 53548-001 | 1800 | item | 187.83 | 0.1044 | 0.1000 | 0.1148 | 1.0000 | 0.1148 | 5% |
| 8.5" x 350 ft. Flexmount double sided tape | Flexcon | DF051521 | 7 | roll | 544.32 | 77.7600 | 0.1000 | 85.5360 | 0.0010 | 0.0862 | 4% |
| 1 µL Disposable micropipette | VWR | 53440-001 | 1000 | item | 62.42 | 0.0624 | 0.1000 | 0.0687 | 1.0000 | 0.0687 | 3% |
| 2 mL Tube | VWR | 53550-790 | 4000 | item | 115.38 | 0.0288 | 0.1000 | 0.0317 | 1.0000 | 0.0317 | 1% |
| Streptavidin alkaline phosphatase | Life Technologies | S921 | 0.5 | mL | 287 | 574.0000 | 0.1000 | 631.4000 | 0.0001 | 0.0316 | 1% |
| 7.5 mL Disposable transfer pipet | VWR | 414004-004 | 5000 | item | 74.38 | 0.0149 | 0.1000 | 0.0164 | 1.0000 | 0.0164 | 1% |
| Transparency film 8.5" x 11" | VWR | 470022-010 | 100 | sheet | 36.18 | 0.3618 | 0.1000 | 0.3980 | 0.0370 | 0.0147 | 1% |
| Phosphate Buffered Saline with 10% Bovine Serum Albumin | Sigma-Aldrich | SRE0036-1L | 1000 | mL | 124 | 0.1240 | 0.1000 | 0.1364 | 0.0999 | 0.0136 | 1% |
| 10x Concentrate, Phosphate Buffered Saline | Sigma-Aldrich | P5493-4L | 4000 | mL | 264 | 0.0660 | 0.1000 | 0.0726 | 0.1199 | 0.0087 | 0% |
| Teknova 10x detection buffer | VWR | 100219-408 | 1000 | mL | 64.89 | 0.0649 | 0.1000 | 0.0714 | 0.0100 | 0.0007 | 0% |
| Tween® 20 | VWR | 97063-874 | 4000 | mL | 57.36 | 0.0143 | 0.1000 | 0.0158 | 0.0001 | 0.0000 | 0% |
| Total | | | | | | | | | | **$2.15** | 100% |

**Table S2**: Packaging cost of single kit

| **Pouching Cost- Single Device** | Unit of Measure | $/Unit | Scrap rate | Price | Devices | Cost/test |
|---|---|---|---|---|---|---|
| Foil Packaging | 1000 | 93 | 10% | 102.30 | 1000 | 0.1023 |
| Desiccant | 10000 | 300 | 10% | 330.00 | 10000 | 0.033 |
| Label Material for Packaging | Roll | 25 | 10% | 27.50 | 350 | 0.07857 |
| **Total Pouching Cost** | | | | | | **$0.21** |

**Table S3**: Overhead cost for production of devices

| **Overhead (Harvard)** | Cost |
|---|---|
| Two FTE | 200,000 |
| 2 FTE Cost/week | 3,846 |
| **Overhead cost/device** | One device |
| 300 Devices/week | **$12.82** |
| **Overhead (Diagnostics For All)** | |
| Two FTE | 200,000 |
| 2 FTE Cost/week | 3,846 |
| **Overhead cost/device** | One device |
| 2000 Devices/week | **$1.92** |
